# Chromosome-level genome sequence data and analysis of the white koji fungus, *Aspergillus luchuensis* mut. *kawachii* IFO 4308

**DOI:** 10.1016/j.dib.2022.107888

**Published:** 2022-02-05

**Authors:** Kazuki Mori, Chihiro Kadooka, Ken Oda, Kayu Okutsu, Yumiko Yoshizaki, Kazunori Takamine, Kosuke Tashiro, Masatoshi Goto, Hisanori Tamaki, Taiki Futagami

**Affiliations:** a Cell Innovator Co., Ltd., 3-1-1 Maidashi, Fukuoka, 812-8582, Japan; b Laboratory of Molecular Gene Technology, Faculty of Agriculture, Kyushu University, 744 Motooka, Nishi-ku, Fukuoka, 819-0395, Japan; c Education and Research Center for Fermentation Studies, Faculty of Agriculture, Kagoshima University, 1-21-24 Korimoto, Kagoshima, 890-0065, Japan; d United Graduate School of Agricultural Sciences, Kagoshima University, 1-21-24 Korimoto, Kagoshima 890-0065, Japan; e National Research Institute of Brewing, 3-7-1 Kagamiyama, Higashi-hiroshima, Hiroshima, 739-0046, Japan; f Department of Applied Biochemistry and Food Science, Faculty of Agriculture, Saga University, Saga 840-8502, Japan

**Keywords:** *Aspergillus luchuensis* mut. *kawachii*, white koji fungus, shochu, chromosome-level genome assembly

## Abstract

*Aspergillus luchuensis* mut. *kawachii* is used primarily in the production of shochu, a traditional Japanese distilled alcoholic beverage. Here, we report the chromosome-level genome sequence of *A. luchuensis* mut. *kawachii* IFO 4308 (NBRC 4308) and a comparison of the sequence with that of *A. luchuensis* RIB2601. The genome of strain IFO 4308 was assembled into nine contigs consisting of eight chromosomes and one mitochondrial DNA segment. The nearly complete genome of strain IFO 4308 comprises 37,287,730 bp with a GC content of 48.85% and 12,664 predicted coding sequences and 267 tRNAs. Comparison of the IFO 4308 and RIB2601 genomes revealed a highly conserved structure; however, the IFO 4308 genome is larger than that of RIB2601, which is primarily attributed to chromosome 5. The genome sequence of IFO 4308 was deposited in DDBJ/ENA/GenBank under accession numbers AP024425–AP024433.

## Specifications Table

**Table:** 

Subject	Biological sciences
Specific subject area	Applied Microbiology, Genomics
Type of data	Genomic sequence Table Figure Supplementary file
How the data were acquired	Whole genome sequencing using Illumina NovaSeq 6000 platform for short reads and Oxford Nanopore Technologies MinION for long reads.
Data format	Raw Assembled/analyzed
Description of data collection	The genomic DNA of strain IFO 4308 was isolated. Raw sequence reads were generated using Illumina NovaSeq 6000 (short reads) and Oxford Nanopore Technologies MinION (long reads). The data were filtered, *de novo* assembled, and annotated using Funannotate pipeline and MFannot.
Data source location	Institution: Kagoshima University City/Town/Region: Kagoshima County: Japan
Data accessibility	The nucleotide sequence of IFO 4308 was deposited in DDBJ/ENA/GenBank under the accession numbers AP024425 (https://www.ncbi.nlm.nih.gov/nuccore/AP024425), AP024426 (https://www.ncbi.nlm.nih.gov/nuccore/AP024426), AP024427 (https://www.ncbi.nlm.nih.gov/nuccore/AP024427), AP024428 (https://www.ncbi.nlm.nih.gov/nuccore/AP024428), AP024429 (https://www.ncbi.nlm.nih.gov/nuccore/AP024429), AP024430 (https://www.ncbi.nlm.nih.gov/nuccore/AP024430), AP024431 (https://www.ncbi.nlm.nih.gov/nuccore/AP024431), AP024432 (https://www.ncbi.nlm.nih.gov/nuccore/AP024432), and AP024433 (https://www.ncbi.nlm.nih.gov/nuccore/AP024433). The nucleotide sequence of IFO 4308 was also deposited in Comprehensive *Aspergillus oryzae* Genome Database (CAoGD) by National Research Institute of Brewing, Japan (https://nribf21.nrib.go.jp/CAoGD/). Raw sequence reads were deposited in the SRA under accession numbers DRX251718 (https://www.ncbi.nlm.nih.gov/sra/DRX251718) and DRX251719 (https://www.ncbi.nlm.nih.gov/sra/DRX251719).

## Value of the Data

• The white koji fungus, *Aspergillus luchuensis* mut. *kawachii*, is used in the production of the traditional Japanese distilled spirit shochu.
• The chromosome-level genome sequence of the white koji fungus can assist shochu brewers and researchers studying koji fungi.
• These data are useful for comparative genomics studies of koji fungi, providing further insights into the genetic background of the white koji fungus that make it superior for use in shochu production.

## 1 Data Description

The white koji fungus, *Aspergillus luchuensis* mut. *kawachii*, is primarily used to produce shochu, a traditional distilled alcoholic beverage indigenous to Japan [1], [2], [3]. The white koji fungus plays an important role in supplying amylolytic enzymes that decompose starch in shochu ingredients, such as rice, barley, buckwheat, and sweet potato. The fungus also secretes large amounts of citric acid that prevent the growth of contaminating microbes during the fermentation process. We previously reported the genome sequence of *A. luchuensis* mut. *kawachii* IFO 4308 (NBRC 4308) [4]. In addition, genome sequences of four other white koji fungi have recently been reported [5]. However, as these sequences were incomplete draft genome assemblies, we conducted a chromosome-level genome analysis of strain IFO 4308.

The nearly complete genome of strain IFO 4308 comprises 37,287,730 bp with a GC content of 48.85% and 12,664 predicted coding sequences and 267 tRNAs. Quality assessment identified 97.7% complete and single-copy, 0.2% complete and duplicate-copy, 0.9% fragmented-copy, and 1.2% missing Benchmarking Universal Single-Copy Orthologs (BUSCOs) [6]. We confirmed that most of the missing BUSCOs were actually present in the genome of IFO 4308. The discrepancy was attributed to technical limitations in gene prediction [6]. Details regarding the chromosomes present in strain IFO 4308 are summarized in Table 1.

**Table 1:** Chromosomes of *A. luchuensis* mut. *kawachii* strain IFO 4308

Location^a^	Accession no.	Size (Mb)	GC%	no. of CDS^b^	no. of rRNA^c^	no. of tRNA
Chr. 1	AP024425.1	6.19	49.5	2,106	NA	47
Chr. 2	AP024426.1	4.96	48.9	1,621	NA	34
Chr. 3	AP024427.1	4.83	49.3	1,636	NA	27
Chr. 4	AP024428.1	3.79	49.2	1,341	15 (72)^d^	17
Chr. 5	AP024429.1	6.27	48.4	2,077	NA	30
Chr. 6	AP024430.1	3.97	48.7	1,386	NA	37
Chr. 7	AP024431.1	3.19	48.2	1,077	NA	12
Chr. 8	AP024432.1	4.05	48.7	1,405	NA	37
MT	AP024433.1	0.03	26.4	15	1	26

a Chr, chromosome; MT, mitochondria.

b CDS, coding DNA sequences.

c NA, not applicable.

d The number of rRNA genes is not clear due to their highly repetitive structure. The number in parentheses indicates the estimated copy number based on the median per-base coverage.

**Fig. 1:**
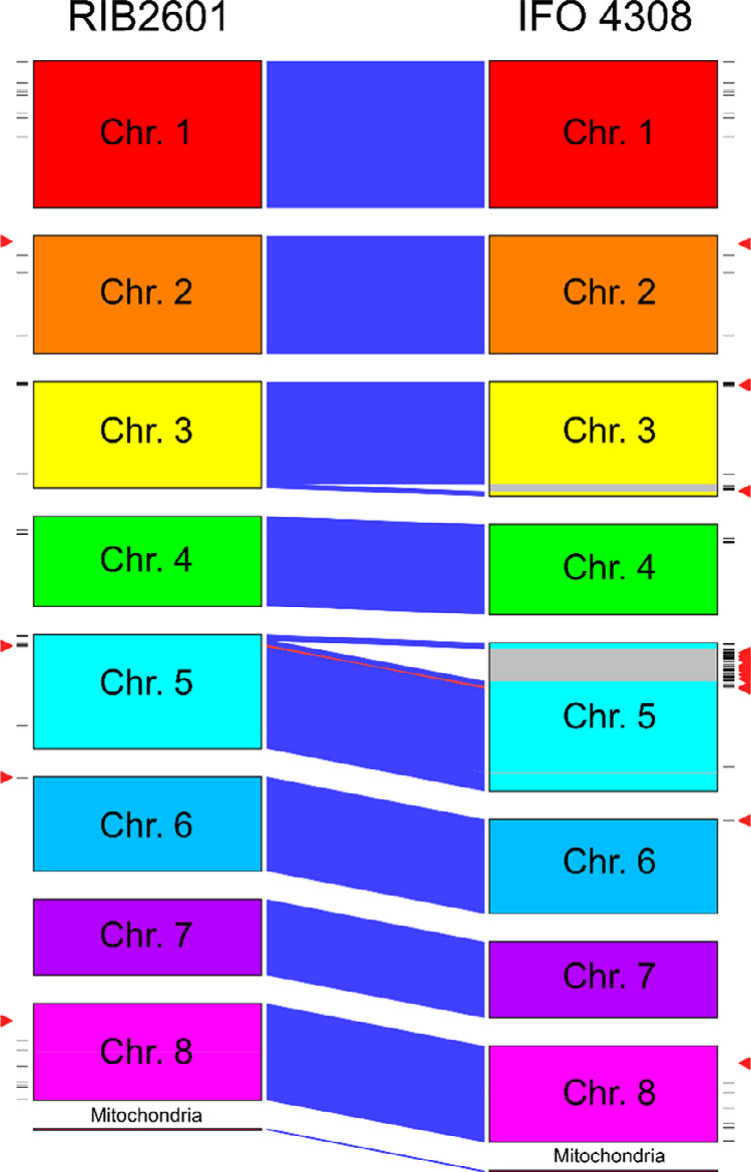
Comparison of the genome structures of *A. luchuensis* mut. *kawachii* strain IFO 4308 and *A. luchuensis* strain RIB2601. The figure was created based on supplementary files. Triangles indicate the locations of genes annotated as reverse transcriptase, whereas lines indicate the locations of repetitive elements annotated as LINEs. Chr, chromosome.

*Aspergillus luchuensis* mut. *kawachii* is an albino mutant of a particular *A. luchuensis* black koji fungus; however, the parent strain of IFO 4308 remains unknown [1], [2], [3],7]. Determination of the nearly complete genome sequence of IFO 4308 enabled us to compare its genomic structure with that of *A. luchuensis* RIB2601, the nearly complete genome of which was sequenced previously [8]. The genome of strain RIB2601 is 35,508,746 bp in size [8], which is smaller than that of strain IFO 4308. Genome comparison indicated a high degree of conservation in the genome structures of strains IFO 4308 and RIB2601, with the larger genome of IFO 4308 primarily attributed to chromosome 5 (Fig. 1). Differences in the genomes could have resulted from transposable elements, such as retrotransposons, because putative reverse transcriptase–encoding genes and long interspersed nuclear elements (LINEs) have been identified in the region specific to IFO 4308 (indicated by triangles and lines in Fig. 1).

## 2 Experimental Design, Materials and Methods

### 2.1 Sequencing and assembly

Strain IFO 4308 was grown in yeast extract-peptone-dextrose medium (2% [wt/vol] glucose, 1% [wt/vol] yeast extract, and 2% [wt/vol] peptone). After cultivation at 30 °C with shaking at 163 rpm for 24 h, mycelia were harvested by filtration. The cell pellet was freeze-dried and ground into powder using a mortar and pestle. DNA was extracted from the mycelial powder using DNAs-ici!-F DNA extraction reagent (Rizo, Inc., Tsukuba, Japan). DNA of strain IFO 4308 was sequenced using a hybrid assembly approach with Oxford Nanopore Technologies (ONT) MinION and Illumina NovaSeq 6000. ONT long reads were used for *de novo* assembly, whereas the Illumina short reads were used for error correction. The genomic library for ONT sequencing was prepared using a Ligation Sequencing Kit (SQK-LSK109) and sequenced via MinION using a flow cell (R9.4.1). Adapter sequences were trimmed using Porechop v0.2.4, and chimeric reads were removed using Yacrd v0.6.1, yielding 1,664,000 ONT reads (mean length, 7,354 bp). The genomic library for Illumina sequencing was prepared using a NEBNext Ultra II DNA Library Prep Kit (E7645) and sequenced via the NovaSeq 6000 using a paired-end sequencing strategy. The Illumina reads were filtered using Fastp v.0.20.1 with default parameters, yielding 42,205,278 reads (mean length, 150 bp). The ONT and Illumina reads provided 328 × and 169 × sequence coverages, respectively. *De novo* assembly of the ONT reads was performed using Canu v.2.0 [9], and the initial assembly and trimmed and corrected ONT reads were reassembled using Flye v2.8-b1674 [10]. Next, several contigs were bridged by contigs generated using MaSuRCA v3.4.2 [11]. The superior metrics were selected based on telomere-to-telomere chromosome assembly. Assemblies were polished using medaka v1.0.3 [12] and pilon v1.23 [13] for ONT reads and pilon v1.23 [13] for Illumina reads. The resulting assembly consisted of nine contigs corresponds to eight chromosomes and one mitochondrial DNA segment. Chromosomes 2, 3, 5, 6, 7, and 8 were generated using only Canu and Flye, whereas chromosomes 1 and 4 were generated via an assembly in which two contigs were bridged using a MaSuRCA contig.

### 2.2 Gene prediction and analysis

The obtained chromosomes and mitochondrial DNA were annotated using the Funannotate v1.8.1 pipeline [14] and MFannot v1.1 [15], respectively. For the Funannotate analysis, the RNA-sequencing (RNA-seq) data for strain IFO 4308 [16] (Sequence Read Archive [SRA] accession numbers SRX9800147 [https://www.ncbi.nlm.nih.gov/sra/SRX9800147] through SRX9800149 [https://www.ncbi.nlm.nih.gov/sra/SRX9800149]) were also used for gene prediction. RNA-seq reads were assembled and mapped using Trinity v2.8.5 [17] and HISAT v2.2.0 [18], respectively, and gene predictions were updated using PASA v2.4.1. Gene products were annotated based on sequence similarity relative to dbCAN2 v9.0 (based on CAZy database v7/30/2020), MEROPS v12.0, MIBiG v1.4, Pfam v33.1, and UniProt v2020_05 databases using antiSMASH v5.1.2, Barrnap v0.9, eggNOG-mapper v1.0.3 (for EggNOG v4.5 database), InterProScan v5.47-82.0, Phobius v1.01, SignalP v4.1, and tRNAscan-SE v2.0.7. Repetitive elements were identified using RepeatMasker v4.1.0 with the Dfam_3.1 and RepBase-20170127 databases [19]. Data from RepeatMasker are provided as supplementary files. Genome assembly and annotation completeness were assessed using BUSCO v5.1.2 with the ascomycota_odb10 (2020-09-10) data set [6]. The genome structures of strains IFO 4308 and RIB2601 were compared using Minimap2 v2.17 [20].

## CRediT Author Statement

**Kazuki Mori:** Conceptualization, Investigation, Writing - Reviewing and Editing; **Chihiro Kadooka:** Investigation, Writing- Reviewing and Editing. **Ken Oda:** Data curation, Visualization, Writing - Reviewing and Editing; **Kayu Okutsu:** Writing - Reviewing and Editing; **Yumiko Yoshizaki:** Writing - Reviewing and Editing; **Kazunori Takamine:** Writing - Reviewing and Editing; **Kosuke Tashiro:** Writing- Reviewing and Editing **Masatoshi Goto:** Data curation, Writing - Reviewing and Editing; **Hisanori Tamaki:** Writing - Reviewing and Editing; **Taiki Futagami:** Supervision, Funding acquisition, Writing-Original draft preparation.

## Declaration of Competing Interest

The authors declare that they have no known competing financial interests or personal relationships that could have appeared to influence the work reported in this paper.

## Appendix Supplementary materials

Image, application 1

Image, application 2
